# β-d-Galacto­pyranosyl-(1→4)–2-amino-2-de­oxy-α-d-gluco­pyran­ose hydro­chloride monohydrate (lactosamine)

**DOI:** 10.1107/S241431462200061X

**Published:** 2022-01-28

**Authors:** Valeri V. Mossine, Steven P. Kelley, Thomas P. Mawhinney

**Affiliations:** a Department of Biochemistry, University of Missouri, Columbia, MO 65211, USA; b Department of Chemistry, University of Missouri, Columbia, MO 65211, USA; Dublin City University, Ireland

**Keywords:** crystal structure, glycosidic bond geometry, Heyns rearrangement, hydrogen bonding

## Abstract

The title compound, C_12_H_24_NO_10_^+^·Cl^−^·H_2_O, (**I**), crystallizes in the monoclinic space group *P*2_1_ and exists as a monohydrate of a monosubstituted ammonium chloride salt, with the reducing carbohydrate portion existing exclusively as the α-pyran­ose tautomer. The glycosidic bond geometry in (**I**) is stabilized by an intra­molecular hydrogen bond and is close to that found in crystalline α-lactose. All heteroatoms except gluco­pyran­ose ring O4 participate in an extensive hydrogen-bonding network, which propagates in all directions in the crystal structure of (**I**).

## Abstract 2

The amino­disaccharide adopts a conformation close to that of the parent α-lactose and is immersed in an extensive hydrogen-bonding network.

## Graphical abstract

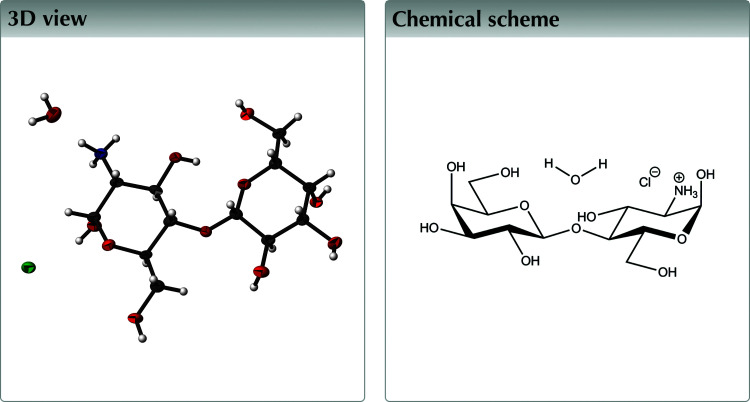

## Structure description

Lactosamine is an important endogenous and food-related glycoepitope that provides for recognition of glycoproteins by both plant and animal β-galactoside-specific lectins, such as tomato lectin (Acarin *et al.*, 1994 ▸) or a family of mammalian galectins (Boscher *et al.*, 2011 ▸; Mossine *et al.*, 2008 ▸). In free and oligomeric form, *N*-acetyl­lactosamine is present in human milk and is believed to participate in the immune protection of infants (Kulinich & Liu, 2016 ▸). Therefore, structural aspects of lactosamine inter­action with carbohydrate-recognizing proteins are of significant inter­est to the biomedical glycobiology field (Seetharaman *et al.*, 1998 ▸; Guardia *et al.*, 2011 ▸). As a part of our research program on the structure and anti-tumorigenic potential of amino­glycoconjugates (Glinskii *et al.*, 2012 ▸; Mossine *et al.*, 2018 ▸), we have prepared a number of 2-amino-2-de­oxy­saccharides, including lactosamine. Although the crystal parameters and hydrogen-bonding geometry of (**I**) were previously reported in a patent (Dekany *et al.*, 2014 ▸), no other structural data have been provided. Here we report details of the mol­ecular geometry of (**I**) and compare it to related disaccharide structures.

**Figure 1:**
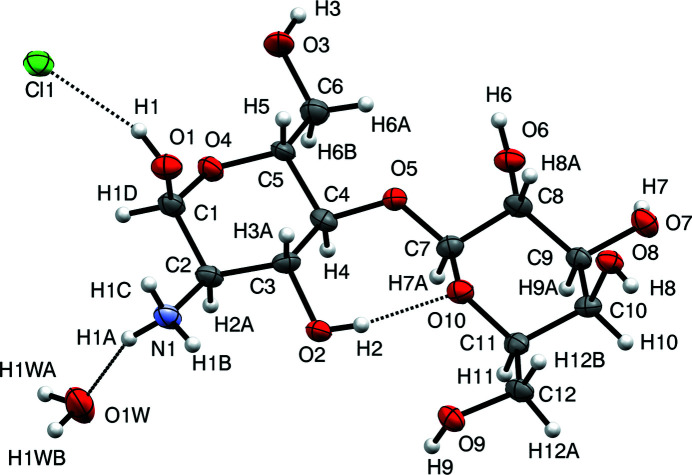
Atomic numbering and displacement ellipsoids at the 50% probability level for (**I**). Hydrogen bonds are shown as dotted lines.

The mol­ecular structure and atomic numbering for the title compound (**I**) are shown in Fig. 1 ▸. Lactosamine is a disaccharide made of the non-reducing β-d-galactoside unit and the d-glucosa­mine portion, which is a reducing end sugar moiety and thus can exist in several tautomeric forms, such as α- and β-pyran­ose, or α- and β-furan­ose. In the crystalline state of (**I**), the d-glucosa­mine residue exists exclusively as the α-pyran­ose anomer, which is also a predominant tautomer in aqueous solutions of lactosamine (Dekany *et al.*, 2014 ▸). The amino group in (**I**) is fully protonated, as would be expected for a hydro­chloride salt. The conformation of the d-glucosa­mine α-pyran­ose ring is a relaxed ^4^ *C*_1_ chair, with puckering parameters *Q*_1_ = 0.579 (8) Å, *θ*_1_ = 1.0 (8)°, and *φ*_1_ = 100 (27)°. The d-galactoside β-pyran­ose ring similarly adopts the ^4^ *C*_1_ conformation, with puckering parameters *Q*_2_ = 0.607 (8) Å, *θ*_2_ = 2.0 (8)°, and *φ*_2_ = 123 (38)°.

The conformation around the β1→4 glycosidic link in disaccharide (**I**) is an important structural characteristic and, for the purpose of the structure comparison, can be conventionally described by the valence angle C4—O5—C7 (also referred to as ‘τ′), torsion angles C4—O5—C7—O10 (‘Φ′) and C3—C4—O5—C7 (‘Ψ’). As can be seen in Table 1 ▸, values of these angles are typical for other Gal-β1→4-Glc disaccharides, with α-lactose monohydrate (Smith *et al.*, 2005 ▸) being conformationally the closest structure to (**I**). It is believed that the O10⋯H—O2 intra­molecular hydrogen bond linking the two carbohydrate units is primarily responsible for stabilization of the spatial arrangement around the glycosidic bond, both in the crystal state and in solutions of Gal-β1→4-Glc di- and oligosaccharides (Imberty *et al.* 1991 ▸). Moreover, this contact may be further stabilized by its involvement in multicenter hydrogen-bonding patterns. For instance, the H2 proton is involved in bifurcated hydrogen bonding with the O5 and O10 acceptors in (**I**) and α-lactose (Tables 2 ▸ and 3 ▸), while in *N*-acetyl­lactosamine (Longchambon *et al.*, 1981 ▸) and *N*-acetyl­lactosyl­amine (Lakshmanan *et al.*, 2001 ▸), additional intra­molecular links between the galacto­pyran­oside and gluco­pyran­ose moieties are represented by the O5⋯H6—O6 and the O9⋯H2—O2 contacts, respectively (Table 2 ▸).

**Figure 2:**
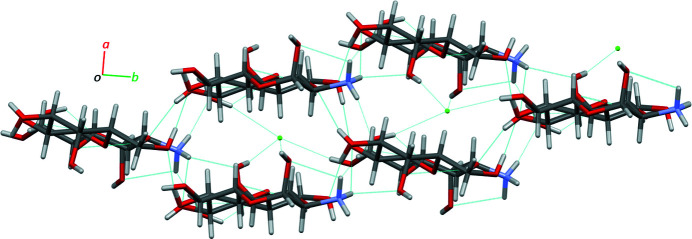
The mol­ecular packing in (**I**) as viewed along the *c* axis. Hydrogen bonds are shown as cyan dotted lines.

**Figure 3:**
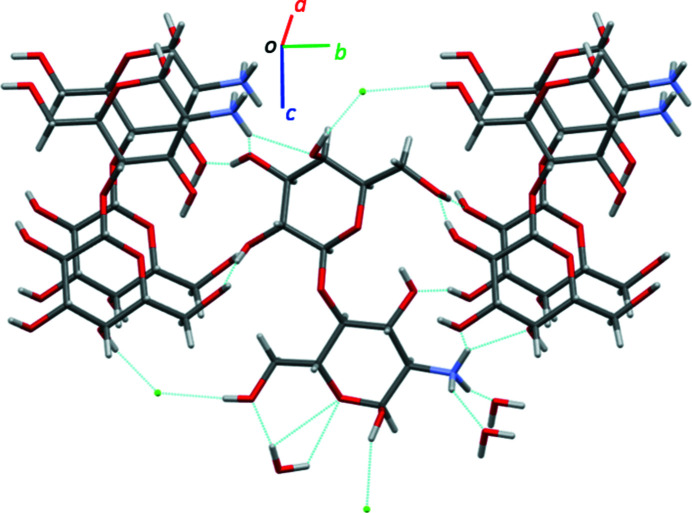
Hydrogen-bonded lactosamine mol­ecular ions, chloride ions, and water mol­ecules surrounding the central lactosamine mol­ecular ion in the crystal structure of (**I**).

**Figure 4:**
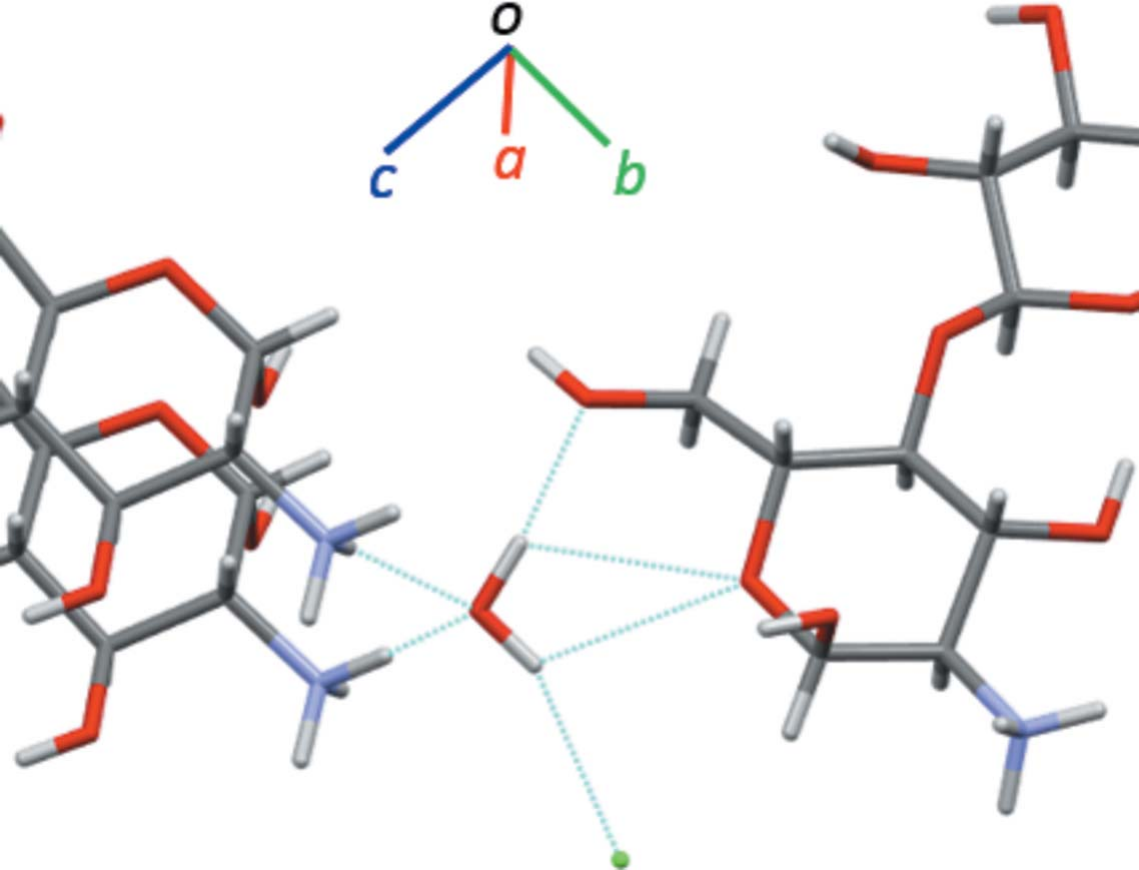
Hydrogen-bonded coordination sphere around the water mol­ecule in the crystal structure of (**I**)

The mol­ecular packing of (**I**) features an extensive inter­molecular hydrogen-bonding network (Table 2 ▸), which propagates in all directions (Fig. 2 ▸). The ammonium groups, chloride ions, and water mol­ecules serve as the hydrogen-bonding network ‘hubs’, each being in short, H-mediated, contact with four or five heteroatoms. For the ammonium group, these are O1, O7, O8, and two different O1*W*; the chloride ions are in contact with O1, O3, O8, and O1*W*; the water mol­ecules are involved in the network by serving as both donors (to Cl1 and O3) and acceptors (to two different H1*A*—N1—H1*C* groups) of strong hydrogen bonding (Table 2 ▸). In this way, each mol­ecule of lactosamine is surrounded by four hydrogen-bonded mol­ecules of lactosamine, three water mol­ecules, and three chloride ions (Fig. 3 ▸); each water mol­ecule coordinates three lactosamines and one chloride (Fig. 4 ▸); every chloride is hydrogen-bonded to three lactosamines and one water as well (Fig. 2 ▸).

## Synthesis and crystallization

The synthesis of (**I**) was performed following a Heyns rearrangement protocol described previously by Wrodnigg & Stütz (1999 ▸). A mixture of 34.2 g (100 mmoles) of d-lactulose and 75 ml (700 mmoles) of benzyl­amine was stirred for 18 h in a screw-capped glass flask at 318 K. The reaction progress was followed by TLC. The excess of benzyl­amine was removed by four successive extractions with benzene (2 L total), the residue was dissolved in 500 ml MeOH containing 20 ml of glacial acetic acid and left for 18 h at room temperature. The reaction mixture was then hydrogenated in the presence of 2.0 g of 10% Pd/C and 5 ml of 80% formic acid, until the reaction was judged complete by TLC. After filtration, the solvents were removed under reduced pressure, a syrupy residue was dissolved in 1.5 L of water and passed through a column charged with 250 ml of ion-exchange resin Amberlite IRN-77 (H^+^-form). The column was washed with water and eluted with 0.2 *M* ammonium acetate. The eluate fractions containing lactosamine were pooled, evaporated to a syrup, re-dissolved in 0.5 L of water and passed through a column filled with 1L of Amberlite IRN-78 (Cl^−^). The eluate fractions containing (**I**) were pooled, evaporated to a syrup, and the syrup was kept at 277 K to produce crystalline material suitable for the X-ray diffraction studies.

## Refinement

Crystal data, data collection and structure refinement details are summarized in Table 4 ▸. The Flack absolute structure parameter determined [0.02 (11) for 729 quotients (Parsons *et al.*, 2013 ▸)] is consistent with the (3*S*,4*R*,5*R*,7*S*,8*R*,9*S*,10*S*,11*R*) configuration, which was assigned for this system on the basis of the known configuration for the starting material d-lactulose (McNaught, 1996 ▸). Data were collected out to 0.80 Å; however, because of the small size of the crystal, most of the high-angle diffraction peaks are effectively indistinguishable from the noise. The inclusion of this high-angle data results in a high value for *R*_int_, and the precision of the bond distances is low (*ca* 0.01 Å) because most of the high-angle data are not usable for refinement.

## Supplementary Material

Crystal structure: contains datablock(s) I. DOI: 10.1107/S241431462200061X/gg4007sup1.cif

Structure factors: contains datablock(s) I. DOI: 10.1107/S241431462200061X/gg4007Isup2.hkl

CCDC reference: 2119923

Additional supporting information:  crystallographic information; 3D view; checkCIF report

## Figures and Tables

**Table 1:** Conformational features (Å, °) of the glycosidic bond in (**I**) and related disaccharide structures with the Gal-β1→4-Glc link

Sugar	Tautomer, conformation	τ	Φ	Ψ	Intra­molecular contacts around glycosidic bond (O⋯H; O⋯O; O⋯H—O)
Gal-β1→4-GlcNH_3_^+^Cl^−^·H_2_O (**I**)*^*a*^*	α-pyran­ose, ^4^ *C*_1_	116.0	−95.2	+90.7	O10⋯H—O2 (1.98; 2.743; 159) O5⋯H—O2 (2.64; 2.964; 106)
Gal-β1→4-GlcNHCOCH_3_·H_2_O (*N*-acetyl­lactosamine, LacNAc·H_2_O)*^*b*^*	α-pyran­ose, ^4^ *C*_1_ *^*b*^*	116.3	−88.1	+97.8	O10⋯H—O2 (1.98; 2.787; 139) O5⋯H—O6 (2.40; 2.868; 122)
LacNAc/ toad galectin*^*c*^*	α-pyran­ose, ^4^C_1_	118.2; 113.6	−66.9; −67.8	+132.4; +132.6	Not reported
LacNAc calculations*^*d*^*	α-pyran­ose, ^4^ *C*_1_ *^*d*^*	117.1	−75	+135	O10⋯H—O2
Gal-β1→4-Glc·H_2_O (α-lactose)*^*e*^*	α-pyran­ose, ^4^ *C*_1_	116.9	−93.4	+95.9	O10⋯H—O2 (2.02; 2.819; 159) O5⋯H—O2 (2.65; 2.992; 106)
Gal-β1→4-Glc (β-lactose)*^*f*^*	β-pyran­ose, ^4^ *C*_1_	116.5	−76.3	+106.4	O10⋯H—O2 (n.d.; 2.707; 101)
Gal-β1→4-GlcNHCOCH_3_·2H_2_O (*N*-acetyl­lactosyl­amine)*^*g*^*	β-pyran­ose, ^4^ *C*_1_	117.4	−89.3	+81.5	O10⋯H—O2 (2.06; 2.767; 144) O9⋯H—O2 (2.45; 3.126; 141)

Notes: (*a*) This work; (*b*) Longchambon *et al.* (1981 ▸); (*c*) Bianchet *et al.* (2000 ▸); (*d*) Imberty *et al.* (1991 ▸); (*e*) Smith *et al.* (2005 ▸); (*f*) Hirotsu & Shimada (1974 ▸); (*g*) Lakshmanan *et al.* (2001 ▸).

**Table 2:** Hydrogen-bond geometry (Å, °)

*D*—H⋯*A*	*D*—H	H⋯*A*	*D*⋯*A*	*D*—H⋯*A*
O1—H1⋯Cl1	0.83 (5)	2.28 (6)	3.075 (6)	163 (9)
O2—H2⋯O10	0.80 (5)	1.98 (6)	2.743 (7)	159 (8)
O3—H3⋯Cl1^i^	0.82 (5)	2.31 (6)	3.130 (7)	172 (9)
O6—H6⋯O9^ii^	0.82 (5)	1.84 (6)	2.654 (8)	171 (9)
O7—H7⋯O2^iii^	0.81 (5)	1.92 (6)	2.697 (8)	158 (9)
O8—H8⋯Cl1^iv^	0.78 (5)	2.32 (6)	3.080 (5)	166 (8)
O9—H9⋯O6^v^	0.83 (5)	1.88 (5)	2.707 (8)	178 (9)
N1—H1*A*⋯O1*W*	0.90 (4)	1.96 (5)	2.819 (9)	159 (7)
N1—H1*B*⋯O7^vi^	0.90 (4)	2.26 (7)	2.862 (8)	124 (6)
N1—H1*B*⋯O8^vi^	0.90 (4)	2.16 (6)	2.922 (8)	142 (7)
N1—H1*C*⋯O1	0.91 (4)	2.34 (8)	2.787 (9)	110 (6)
N1—H1*C*⋯O1*W* ^vii^	0.91 (4)	2.35 (6)	3.162 (11)	149 (7)
O1*W*—H1*WA*⋯O3^viii^	0.90 (6)	1.85 (7)	2.746 (8)	170 (10)
O1*W*—H1*WB*⋯Cl1^ix^	0.89 (6)	2.50 (7)	3.335 (7)	156 (9)

Symmetry codes: (i) 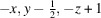 ; (ii) 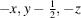 ; (iii) 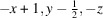 ; (iv) 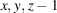 ; (v) 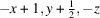 ; (vi) 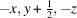 ; (vii) 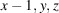 ; (viii) 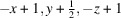 ; (ix) 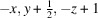 .

**Table 3:** Additional *D*—H⋯*A* contacts (Å, °)

*D*—H⋯*A*	*D*—H	H⋯*A*	*D*⋯*A*	*D*—H⋯*A*
O2—H2⋯O5	0.80 (7)	2.64 (8)	2.964 (8)	106 (6)
N1—H1*B*⋯O2	0.90 (6)	2.55 (7)	2.855 (9)	101 (5)
O7—H7⋯O6	0.81 (8)	2.63 (8)	2.847 (8)	97 (8)
C2—H2*A*⋯O1^i^	0.98	2.34	3.199 (10)	146
C9—H9*A*⋯O8^i^	0.98	2.58	3.309 (9)	132
C10—H10⋯Cl1^ii^	0.98	2.82	3.741 (9)	157

Symmetry codes: (i) *x* + 1, *y*, *z*; (ii) *x* + 1, *y*, *z* − 1.

**Table 4:** Experimental details

Crystal data	
Chemical formula	C_12_H_24_NO_10_^+^·Cl^−^·H_2_O
*M*_r_	395.79
Crystal system, space group	Monoclinic, *P*2_1_
Temperature (K)	273
*a*, *b*, *c* (Å)	4.785 (4), 13.523 (11), 13.254 (11)
β (°)	93.940 (9)
*V* (Å^3^)	855.5 (12)
*Z*	2
Radiation type	Mo *K*α
μ (mm^−1^)	0.28
Crystal size (mm)	0.08 × 0.05 × 0.01

Data collection	
Diffractometer	Bruker APEXII area detector
Absorption correction	Multi-scan (*AXScale*; Bruker, 2016 ▸)
*T*_min_, *T*_max_	0.483, 0.746
No. of measured, independent and observed [*I* > 2σ(*I*)] reflections	11475, 3787, 2216
*R*_int_	0.133
(sin θ/λ)_max_ (Å^−1^)	0.643

Refinement	
*R*[*F* ^2^ > 2σ(*F* ^2^)], *wR*(*F* ^2^), *S*	0.066, 0.131, 1.01
No. of reflections	3787
No. of parameters	262
No. of restraints	26
H-atom treatment	H atoms treated by a mixture of independent and constrained refinement
Δρ_max_, Δρ_min_ (e Å^−3^)	0.44, −0.37
Absolute structure	Flack *x* determined using 729 quotients [(*I* ^+^)−(*I* ^−^)]/[(*I* ^+^)+(*I* ^−^)] (Parsons *et al.*, 2013 ▸)
Absolute structure parameter	0.02 (11)

Computer programs: *APEX3* and *SAINT* (Bruker, 2016 ▸), *SHELXS* (Sheldrick, 2008 ▸), *SHELXL2017/1* (Sheldrick, 2015 ▸), and *OLEX2* (Dolomanov *et al.*, 2009 ▸).

## full crystallographic data

### Crystal data

**Table:**

C_12_H_24_NO_10_^+^·Cl^−^·H_2_O	*F*(000) = 420
*M**_r_* = 395.79	*D*_x_ = 1.536 Mg m^−^^3^
Monoclinic, *P*2_1_	Mo *K*α radiation, λ = 0.71073 Å
*a* = 4.785 (4) Å	Cell parameters from 1276 reflections
*b* = 13.523 (11) Å	θ = 3.0–20.6°
*c* = 13.254 (11) Å	µ = 0.28 mm^−^^1^
β = 93.940 (9)°	*T* = 273 K
*V* = 855.5 (12) Å^3^	Plate, colourless
*Z* = 2	0.08 × 0.05 × 0.01 mm

### Data collection

**Table:**

Bruker APEXII area detector diffractometer	2216 reflections with *I* > 2σ(*I*)
Radiation source: Sealed Source Mo with TRIUMPH optics	*R*_int_ = 0.133
ω and phi scans	θ_max_ = 27.2°, θ_min_ = 1.5°
Absorption correction: multi-scan (*AXScale*; Bruker, 2016)	*h* = −6→6
*T*_min_ = 0.483, *T*_max_ = 0.746	*k* = −17→17
11475 measured reflections	*l* = −17→16
3787 independent reflections	

### Refinement

**Table:**

Refinement on *F*^2^	Secondary atom site location: difference Fourier map
Least-squares matrix: full	Hydrogen site location: mixed
*R*[*F*^2^ > 2σ(*F*^2^)] = 0.066	H atoms treated by a mixture of independent and constrained refinement
*wR*(*F*^2^) = 0.131	*w* = 1/[σ^2^(*F*_o_^2^) + (0.0475*P*)^2^] where *P* = (*F*_o_^2^ + 2*F*_c_^2^)/3
*S* = 1.01	(Δ/σ)_max_ < 0.001
3787 reflections	Δρ_max_ = 0.44 e Å^−^^3^
262 parameters	Δρ_min_ = −0.37 e Å^−^^3^
26 restraints	Absolute structure: Flack *x* determined using 729 quotients [(*I*^+^)-(*I*^-^)]/[(*I*^+^)+(*I*^-^)] (Parsons *et al.*, 2013)
Primary atom site location: structure-invariant direct methods	Absolute structure parameter: 0.02 (11)

### Special details

**Table:**

Geometry. All esds (except the esd in the dihedral angle between two l.s. planes) are estimated using the full covariance matrix. The cell esds are taken into account individually in the estimation of esds in distances, angles and torsion angles; correlations between esds in cell parameters are only used when they are defined by crystal symmetry. An approximate (isotropic) treatment of cell esds is used for estimating esds involving l.s. planes.
Refinement. Hydroxy and nitrogen-bound H atoms were located in difference-Fourier analyses and were allowed to refine fully. Other H atoms were placed at calculated positions and treated as riding. All chemically equivalent N—H and O—H bond distances were restrained to be equal within 0.05 Å.

### Fractional atomic coordinates and isotropic or equivalent isotropic displacement parameters (Å^2^)

**Table:**

	*x*	*y*	*z*	*U*_iso_*/*U*_eq_	
Cl1	−0.3280 (4)	0.75359 (14)	0.61589 (14)	0.0286 (5)	
O1	−0.1394 (11)	0.7867 (4)	0.4014 (4)	0.0272 (13)	
H1	−0.195 (16)	0.765 (7)	0.455 (5)	0.041*	
O1W	0.6240 (15)	1.0329 (4)	0.4269 (5)	0.0462 (19)	
H1WA	0.71 (2)	1.021 (8)	0.488 (6)	0.069*	
H1WB	0.58 (2)	1.096 (5)	0.433 (8)	0.069*	
O2	0.2355 (12)	0.8551 (4)	0.1351 (4)	0.0260 (14)	
H2	0.186 (17)	0.830 (6)	0.082 (5)	0.039*	
O3	0.1487 (12)	0.4761 (4)	0.3861 (4)	0.0341 (14)	
H3	0.18 (2)	0.416 (4)	0.387 (7)	0.051*	
O4	0.2482 (10)	0.6815 (4)	0.3919 (4)	0.0253 (13)	
O5	0.1019 (10)	0.6413 (4)	0.1208 (4)	0.0223 (12)	
O6	0.3161 (10)	0.4684 (4)	0.0307 (4)	0.0263 (12)	
H6	0.175 (14)	0.446 (6)	0.055 (6)	0.039*	
O7	0.3237 (11)	0.4742 (4)	−0.1838 (4)	0.0266 (13)	
H7	0.431 (16)	0.434 (5)	−0.157 (6)	0.040*	
O8	−0.0409 (10)	0.6292 (4)	−0.2120 (4)	0.0229 (12)	
H8	−0.091 (17)	0.658 (6)	−0.261 (5)	0.034*	
O9	0.1635 (11)	0.9157 (4)	−0.1046 (4)	0.0255 (13)	
H9	0.321 (13)	0.933 (6)	−0.082 (6)	0.038*	
O10	0.1420 (10)	0.7280 (3)	−0.0250 (4)	0.0226 (13)	
N1	0.1495 (15)	0.9460 (5)	0.3249 (5)	0.0267 (17)	
H1A	0.270 (14)	0.978 (6)	0.369 (5)	0.040*	
H1B	0.104 (16)	0.980 (6)	0.268 (4)	0.040*	
H1C	−0.023 (11)	0.946 (6)	0.350 (6)	0.040*	
C1	0.1489 (16)	0.7791 (5)	0.4024 (6)	0.0250 (18)	
H1D	0.231817	0.807016	0.465797	0.030*	
C2	0.2409 (16)	0.8418 (5)	0.3134 (6)	0.0227 (18)	
H2A	0.445983	0.840893	0.315433	0.027*	
C3	0.1292 (16)	0.7968 (5)	0.2135 (6)	0.0228 (18)	
H3A	−0.076034	0.799022	0.208350	0.027*	
C4	0.2270 (15)	0.6906 (6)	0.2095 (6)	0.0224 (18)	
H4	0.431371	0.689282	0.207687	0.027*	
C5	0.1419 (16)	0.6315 (6)	0.3013 (5)	0.0226 (17)	
H5	−0.062765	0.627481	0.300031	0.027*	
C6	0.2641 (17)	0.5292 (6)	0.3051 (6)	0.030 (2)	
H6A	0.219239	0.495436	0.241483	0.036*	
H6B	0.466372	0.532736	0.316291	0.036*	
C7	0.2534 (15)	0.6458 (6)	0.0341 (5)	0.0222 (17)	
H7A	0.453220	0.655704	0.052771	0.027*	
C8	0.2081 (16)	0.5518 (5)	−0.0258 (6)	0.0217 (17)	
H8A	0.007235	0.542472	−0.042707	0.026*	
C9	0.3596 (16)	0.5604 (5)	−0.1230 (5)	0.0204 (17)	
H9A	0.559991	0.570369	−0.105595	0.024*	
C10	0.2464 (15)	0.6483 (6)	−0.1834 (6)	0.0213 (17)	
H10	0.350340	0.656234	−0.244111	0.026*	
C11	0.2853 (15)	0.7402 (6)	−0.1168 (5)	0.0220 (17)	
H11	0.485554	0.750477	−0.099407	0.026*	
C12	0.1643 (16)	0.8313 (5)	−0.1692 (6)	0.0241 (18)	
H12A	0.272384	0.846504	−0.226525	0.029*	
H12B	−0.026310	0.817433	−0.194879	0.029*	

### Atomic displacement parameters (Å^2^)

**Table:**

	*U*^11^	*U*^22^	*U*^33^	*U*^12^	*U*^13^	*U*^23^
Cl1	0.0323 (11)	0.0230 (11)	0.0302 (10)	−0.0014 (9)	−0.0006 (8)	0.0045 (10)
O1	0.027 (3)	0.023 (3)	0.032 (4)	0.002 (2)	0.004 (3)	0.004 (3)
O1W	0.075 (5)	0.027 (4)	0.034 (4)	−0.003 (3)	−0.012 (3)	−0.002 (3)
O2	0.035 (3)	0.021 (3)	0.022 (3)	−0.010 (2)	−0.001 (3)	0.001 (2)
O3	0.056 (4)	0.016 (3)	0.030 (3)	−0.003 (3)	0.004 (3)	0.007 (3)
O4	0.033 (3)	0.017 (3)	0.026 (3)	−0.002 (2)	−0.001 (3)	0.000 (2)
O5	0.027 (3)	0.021 (3)	0.020 (3)	−0.005 (2)	0.002 (2)	0.001 (2)
O6	0.025 (3)	0.021 (3)	0.033 (3)	0.001 (3)	0.005 (2)	0.006 (3)
O7	0.032 (3)	0.016 (3)	0.031 (3)	0.005 (2)	−0.001 (2)	−0.001 (3)
O8	0.025 (3)	0.019 (3)	0.024 (3)	−0.001 (2)	−0.001 (2)	0.004 (2)
O9	0.026 (3)	0.019 (3)	0.032 (3)	0.000 (2)	0.001 (3)	−0.003 (3)
O10	0.024 (3)	0.017 (3)	0.026 (3)	0.003 (2)	0.003 (2)	0.003 (2)
N1	0.035 (4)	0.021 (4)	0.023 (4)	−0.005 (3)	−0.006 (3)	0.004 (3)
C1	0.030 (5)	0.018 (4)	0.026 (4)	−0.002 (3)	−0.001 (3)	0.003 (3)
C2	0.025 (4)	0.016 (4)	0.026 (5)	−0.003 (3)	−0.001 (4)	0.004 (3)
C3	0.023 (4)	0.021 (4)	0.024 (4)	−0.002 (3)	0.002 (3)	0.008 (3)
C4	0.020 (4)	0.019 (4)	0.029 (5)	−0.004 (3)	0.000 (3)	0.000 (4)
C5	0.029 (4)	0.016 (4)	0.023 (4)	−0.009 (3)	−0.001 (3)	0.002 (3)
C6	0.037 (5)	0.023 (5)	0.028 (5)	−0.003 (4)	0.000 (4)	0.003 (4)
C7	0.023 (4)	0.021 (4)	0.023 (4)	0.001 (3)	0.002 (3)	0.004 (4)
C8	0.024 (4)	0.015 (4)	0.026 (5)	0.003 (3)	−0.002 (3)	0.005 (3)
C9	0.021 (4)	0.016 (4)	0.024 (4)	0.002 (3)	0.002 (3)	−0.001 (4)
C10	0.020 (4)	0.019 (4)	0.025 (4)	−0.002 (3)	0.004 (3)	0.003 (3)
C11	0.021 (4)	0.020 (4)	0.027 (4)	−0.001 (3)	0.007 (3)	−0.002 (4)
C12	0.026 (4)	0.014 (4)	0.032 (5)	0.001 (3)	0.004 (4)	0.000 (4)

### Geometric parameters (Å, º)

**Table:**

O1—C1	1.382 (9)	N1—H1C	0.91 (4)
O1—H1	0.83 (5)	C1—C2	1.541 (10)
O1W—H1WA	0.90 (6)	C1—H1D	0.9800
O1W—H1WB	0.89 (6)	C2—C3	1.521 (10)
O2—C3	1.426 (9)	C2—H2A	0.9800
O2—H2	0.80 (5)	C3—C4	1.512 (10)
O3—C6	1.434 (10)	C3—H3A	0.9800
O3—H3	0.82 (5)	C4—C5	1.533 (10)
O4—C1	1.414 (9)	C4—H4	0.9800
O4—C5	1.440 (8)	C5—C6	1.502 (11)
O5—C7	1.402 (9)	C5—H5	0.9800
O5—C4	1.445 (9)	C6—H6A	0.9700
O6—C8	1.431 (9)	C6—H6B	0.9700
O6—H6	0.82 (5)	C7—C8	1.507 (10)
O7—C9	1.420 (9)	C7—H7A	0.9800
O7—H7	0.81 (5)	C8—C9	1.526 (10)
O8—C10	1.424 (9)	C8—H8A	0.9800
O8—H8	0.78 (5)	C9—C10	1.513 (10)
O9—C12	1.426 (9)	C9—H9A	0.9800
O9—H9	0.83 (5)	C10—C11	1.528 (10)
O10—C7	1.441 (8)	C10—H10	0.9800
O10—C11	1.447 (8)	C11—C12	1.510 (10)
N1—C2	1.486 (10)	C11—H11	0.9800
N1—H1A	0.90 (4)	C12—H12A	0.9700
N1—H1B	0.90 (4)	C12—H12B	0.9700
			
C1—O1—H1	110 (6)	C6—C5—H5	109.6
H1WA—O1W—H1WB	101 (9)	C4—C5—H5	109.6
C3—O2—H2	108 (6)	O3—C6—C5	108.5 (7)
C6—O3—H3	115 (7)	O3—C6—H6A	110.0
C1—O4—C5	114.7 (5)	C5—C6—H6A	110.0
C7—O5—C4	116.0 (5)	O3—C6—H6B	110.0
C8—O6—H6	102 (6)	C5—C6—H6B	110.0
C9—O7—H7	105 (6)	H6A—C6—H6B	108.4
C10—O8—H8	112 (6)	O5—C7—O10	106.7 (5)
C12—O9—H9	114 (6)	O5—C7—C8	109.3 (6)
C7—O10—C11	111.5 (5)	O10—C7—C8	109.3 (5)
C2—N1—H1A	110 (5)	O5—C7—H7A	110.5
C2—N1—H1B	117 (5)	O10—C7—H7A	110.5
H1A—N1—H1B	114 (7)	C8—C7—H7A	110.5
C2—N1—H1C	109 (6)	O6—C8—C7	110.8 (6)
H1A—N1—H1C	109 (8)	O6—C8—C9	109.1 (6)
H1B—N1—H1C	97 (7)	C7—C8—C9	108.7 (6)
O1—C1—O4	114.2 (6)	O6—C8—H8A	109.4
O1—C1—C2	106.8 (6)	C7—C8—H8A	109.4
O4—C1—C2	108.8 (6)	C9—C8—H8A	109.4
O1—C1—H1D	109.0	O7—C9—C10	108.6 (6)
O4—C1—H1D	109.0	O7—C9—C8	111.8 (6)
C2—C1—H1D	109.0	C10—C9—C8	109.5 (6)
N1—C2—C3	112.4 (6)	O7—C9—H9A	109.0
N1—C2—C1	109.9 (6)	C10—C9—H9A	109.0
C3—C2—C1	110.2 (6)	C8—C9—H9A	109.0
N1—C2—H2A	108.1	O8—C10—C9	107.6 (6)
C3—C2—H2A	108.1	O8—C10—C11	112.3 (6)
C1—C2—H2A	108.1	C9—C10—C11	107.9 (6)
O2—C3—C4	111.9 (7)	O8—C10—H10	109.7
O2—C3—C2	106.9 (6)	C9—C10—H10	109.7
C4—C3—C2	108.6 (6)	C11—C10—H10	109.7
O2—C3—H3A	109.8	O10—C11—C12	106.9 (6)
C4—C3—H3A	109.8	O10—C11—C10	110.3 (6)
C2—C3—H3A	109.8	C12—C11—C10	111.7 (6)
O5—C4—C3	110.8 (6)	O10—C11—H11	109.3
O5—C4—C5	106.7 (6)	C12—C11—H11	109.3
C3—C4—C5	111.6 (6)	C10—C11—H11	109.3
O5—C4—H4	109.3	O9—C12—C11	113.2 (6)
C3—C4—H4	109.3	O9—C12—H12A	108.9
C5—C4—H4	109.3	C11—C12—H12A	108.9
O4—C5—C6	106.8 (6)	O9—C12—H12B	108.9
O4—C5—C4	108.6 (6)	C11—C12—H12B	108.9
C6—C5—C4	112.5 (7)	H12A—C12—H12B	107.7
O4—C5—H5	109.6		

### Hydrogen-bond geometry (Å, º)

**Table:**

*D*—H···*A*	*D*—H	H···*A*	*D*···*A*	*D*—H···*A*
O1—H1···Cl1	0.83 (5)	2.28 (6)	3.075 (6)	163 (9)
O2—H2···O10	0.80 (5)	1.98 (6)	2.743 (7)	159 (8)
O3—H3···Cl1^i^	0.82 (5)	2.31 (6)	3.130 (7)	172 (9)
O6—H6···O9^ii^	0.82 (5)	1.84 (6)	2.654 (8)	171 (9)
O7—H7···O2^iii^	0.81 (5)	1.92 (6)	2.697 (8)	158 (9)
O8—H8···Cl1^iv^	0.78 (5)	2.32 (6)	3.080 (5)	166 (8)
O9—H9···O6^v^	0.83 (5)	1.88 (5)	2.707 (8)	178 (9)
N1—H1*A*···O1*W*	0.90 (4)	1.96 (5)	2.819 (9)	159 (7)
N1—H1*B*···O7^vi^	0.90 (4)	2.26 (7)	2.862 (8)	124 (6)
N1—H1*B*···O8^vi^	0.90 (4)	2.16 (6)	2.922 (8)	142 (7)
N1—H1*C*···O1	0.91 (4)	2.34 (8)	2.787 (9)	110 (6)
N1—H1*C*···O1*W*^vii^	0.91 (4)	2.35 (6)	3.162 (11)	149 (7)
O1*W*—H1*WA*···O3^viii^	0.90 (6)	1.85 (7)	2.746 (8)	170 (10)
O1*W*—H1*WB*···Cl1^ix^	0.89 (6)	2.50 (7)	3.335 (7)	156 (9)

Symmetry codes: (i) −*x*, *y*−1/2, −*z*+1; (ii) −*x*, *y*−1/2, −*z*; (iii) −*x*+1, *y*−1/2, −*z*; (iv) *x*, *y*, *z*−1; (v) −*x*+1, *y*+1/2, −*z*; (vi) −*x*, *y*+1/2, −*z*; (vii) *x*−1, *y*, *z*; (viii) −*x*+1, *y*+1/2, −*z*+1; (ix) −*x*, *y*+1/2, −*z*+1.
